# Origins of Injection-Site Sarcomas in Cats: The Possible Role of Chronic Inflammation—A Review

**DOI:** 10.5402/2011/210982

**Published:** 2011-04-12

**Authors:** Kevin N. Woodward

**Affiliations:** ^1^Technology Sciences (Europe) Limited, Concordia House, St James Business Park, Grimbald Crag Court, Knaresborough, North Yorkshire, HG5 8QB, UK; ^2^Intervet/Schering-Plough Animal Health, Breakspear Road South, Harefield, Uxbridge, Middlesex, UB9 6LS, UK

## Abstract

The etiology of feline injection-site sarcomas remains obscure. Sarcomas and other tumors are known to be associated with viral infections in humans and other animals, including cats. However, the available evidence suggests that this is not the case with feline injection-site sarcomas. These tumors have more in common with sarcomas noted in experimental studies with laboratory animals where foreign materials such as glass, plastics, and metal are the causal agent. Tumors arising with these agents are associated with chronic inflammation at the injection or implantation sites. Similar tumors have been observed, albeit infrequently, at microchip implantation sites, and these also are associated with chronic inflammation. It is suggested that injection-site sarcomas in cats may arise at the administration site as a result of chronic inflammation, possibly provoked by adjuvant materials, with subsequent DNA damage, cellular transformation, and clonal expansion. However, more fundamental research is required to elucidate the mechanisms involved.

## 1. Introduction

The majority of cancers of humans and other animals are carcinomas. These are tumours of epidermal origin that can invade and metastasise and which may ultimately prove fatal. Another group of malignant tumours are the sarcomas. These are tumours, probably of mesenchymal origin that, like carcinomas, are also locally invasive and have the ability to metastasise and again, are also found in humans [1–4]. In cats and dogs, a variety of soft tissue sarcomas may arise spontaneously including fibrosarcoma, myxosarcoma, liposarcoma, neurofibrosarcoma, malignant Schwannoma, rhabdomyosarcoma, and leiomyosarcoma [5–7]. Although these tumours are amenable to treatment, local recurrence is common after resection and they are locally invasive; those with a diameter of >5 cm show a poor response to radiotherapy and to chemotherapy [3, 7–17].

One of the results of veterinary pharmacovigilance has been the recognition of the development of sarcomas at the injection site in cats following routine vaccination and occasionally, following administration of pharmaceutical products [18–29]. These tumors are usually fibrosarcomas. As with spontaneous fibrosarcomas, many of those associated with injection sites show a variety of morphological and subcellular abnormalities [30–34]. They are frequently fast growing and aggressive and may spread locally and aggressively along fascial planes and metastasise to remote sites [12, 35–41]. These tumors tend to consist of elongated cells supported by a fibrovascular stroma. Areas of necrosis are reported to be common along with infiltrates of lymphocytes, macrophages, and multinucleate giant cells, and there are higher mitotic rates than are noted in noninjection site sarcomas [18, 31, 32, 42, 43]. Like spontaneously occurring sarcomas, they are challenging to treat [10, 16, 37, 44–49]. There have been a number of case reports of such tumours following vaccination [19, 20, 23, 50–55]. Additionally, there have been reports on rhabdomyosarcoma, malignant fibrous histiocytoma, chondroma, sarcoma, myofibrosarcoma, and liposarcoma developing at the injection site in cats [43, 50, 56–58]. As with spontaneously occurring tumors, these injection-associated sarcomas are amenable to treatment. This normally involves radiotherapy and chemotherapy with various agents including carboplatin, doxorubicin (including doxil, a doxorubicin hydrochloride liposome formulation), cyclophosphamide and vincristine as well as immunotherapy; such treatments provide remission but many cats will finally succumb to the disease [8, 44, 59–65].

It should be emphasised that the frequency of these injection-site tumours is rare [66–69]. For example, the prevalence in the US was estimated to be 0.00021 cases/cat visit or 2.1 cases per 10 000 cat visits for 1992 [66]. Moreover, the undoubted benefits of vaccination against serious and frequently fatal infectious diseases clearly outweigh the risks associated with vaccination. Nevertheless, the frequency of these lesions, or their diagnostic recognition, does appear to be increasing [24, 25, 27, 38, 42, 43, 53, 56, 57, 69–82]. For example, in the United Kingdom, the number of reports of injection-site sarcomas associated with vaccines submitted to the regulatory agency, the Veterinary Medicines Directorate (VMD) has risen over the period from 2001 to 2008 [83–91]. In 2009, the latest year for which figures were available, 40 reports were submitted to the VMD [91].

Despite considerable research over the last few decades since the phenomenon was first noted, there is currently no plausible explanation for the development of fibrosarcomas in cats at the injection site.

## 2. Possible Etiologies

Virus-associated cancers, including sarcomas, are well-known tumours of cats but where these have been sought in injection-site associated lesions, they have not been found [32, 92, 93]. Other explanations must be sought. In a review of human sarcomas, one authority notes that there is case history evidence for the development of sarcomas at the site of repeated trauma or foreign body implants [1]. He goes on to say that this is nonsense because it assumes the pre-existence of a dormant sarcoma at the injury site. So the roles of repeated trauma or of foreign bodies in the development of fibrosarcomas can be dismissed. Or can they? Other evidence notably from toxicology and experimental pathology might suggest otherwise.

Grasso and Golberg demonstrated the induction of sarcomas in rats after the subcutaneous injection of certain food colourings [94]. Some food colorants such as Patent Blue V produced no appreciable tissue reaction while others including Brilliant Blue FCF led to an intense macrophage response with macrophage necrosis and proliferation of fibroblasts. Other food colourings produced the development of collagenous material with a “persistent fibroblastic reaction.” The colorants which produced these tissue reactions were also shown to result in fibrosarcomas at the injection sites despite the fact that the substances tested were not classical chemical carcinogens.

Similar findings in rats and mice have been noted with other substances some of which were carcinogens while others, notably iron derivatives, some polymeric rubber additives, polyvinyl pyrrolidones, vitamin E, soya oil, and Tween 80, were clearly not [95–107]. Leaving frank carcinogens aside, it was noted that the otherwise biologically inert materials which did lead to intense tissue reactions and injection site sarcomas were those which possessed surface activity, acidic pH or precipitated at the site of injection [103, 107–110].

Furthermore, subcutaneous implanting of solid materials including various polymers, including Cellophane, plastic films, hydrophilic Millipore discs, Bakelite, Teflon, glass, and particulate plastic materials, has resulted in sarcomagenesis in rats and mice [111–134]. As with the chemicals discussed above, the formation of sarcomas was preceded by fibrosis and the production of collagenous material [123, 125, 134]. It appears that there may be a biphasic response with an initial phase involving cellular proliferation and tissue infiltration and later, fibrosis [135, 136]. The carcinogenic process is thought to involve inflammation, with reactive oxygen species playing an important role in the ensuing genetic damage [137, 138].

Together, these findings gave rise to the terms foreign-body sarcomas or more broadly, solid state carcinogenesis [139]. They also gave rise to concerns over implants, prostheses, and medical devices used in human medicine but the available evidence does not suggest these are associated with undue risks, certainly from the point of view of local fibrosarcoma induction [140–150] although there have been cases of various sarcomas including osteosarcoma, Ewing's sarcoma, and angiosarcoma associated with prostheses and vascular grafts [151–153]. Cancers have been reported at the sites of shrapnel injuries where fragments have remained for several years [154–156]. Moreover, there have been reports that intramuscular injections of various drugs, but notably iron compounds, have given rise to granulomas, as well as sarcomas and other tumours at the injection site [157–165]. Unfortunately, the diverse nature of the drugs (iron dextran, diclofenac, insulin zinc (Lente), and penicillin) and the fact that some patients were receiving immunosuppressive therapy make the available data difficult to interpret. Any risks, especially with iron compounds, are likely to be very small [166, 167]. Nevertheless, all of these findings, and notably those from animal studies, raise questions on the role of inflammation in the induction of tumors.

## 3. The Possible Role of Chronic Inflammation

Vaccines are composed of antigenic materials dissolved or suspended in solution. The antigens are unlikely to be carcinogenic and solvent systems are carefully chosen for safety. Many inactivated vaccines also contain adjuvants or immunopotentiators [168–170]. Veterinary vaccines are subjected to rigorous safety testing and evaluation prior to authorisation [171, 172] but vaccination is not without risks in humans, and in animals although these risks are low [29, 173–178].

Many inactivated veterinary vaccines contain aluminium hydroxide, an insoluble aluminium compound, or other aluminium derivatives as adjuvant [170] and in companion animals such as dogs and cats, these vaccines are frequently given subcutaneously. Could this aluminium material be behaving as a foreign-body carcinogen in the cat? There is some evidence for this.

The cat certainly seems susceptible to fibrosarcomas as the data on vaccines already reviewed here demonstrates. Moreover, fibrosarcomas have been reported in cats at the site of microchip implantation [179] and at the site of a nonabsorbale suture [180]. Interestingly, although the injection site-associated sarcoma story has largely focussed on cats, they have also been noted in the dog [181] while, sarcomas adjacent to microchip implantation have also been observed in this species [182, 183]. Microchips have been shown to elicit sarcomas in mice [184]. An osteosarcoma has been reported at the site of a retained surgical sponge in the dog [185]. Aluminium has been detected in an injection-site sarcoma resected from a cat [32, 92]. Using electron probe microanalysis, aluminium was detected in a number of injection-site sarcomas obtained from cats [186]. 

These observations do not provide conclusive proof that aluminium is involved in foreign-body carcinogenesis in the cat (or dog). However, aluminium is relatively inert as indeed are microchips and many of the plastics, films, and metals employed in the experimental studies discussed above. So, what possible mechanism could be involved?

Unfortunately, there are not too many (known) options. The immediate reaction to a foreign body is acute inflammation, a protective mechanism which aims to remove the foreign body for example, by the killing and lysis of bacterial cells. It is of short duration and characterised by exudation of fluid and plasma proteins and migration of leukocytes. It involves a number of humoral and cellular mediators including histamine, serotonin, prostaglandins, cytokines, nitric oxide, and reactive oxygen species, and following removal of the offending object, resolution occurs.

If the foreign body cannot be removed, for example, because it is insoluble—a metal particle, glass fragment, plastic, or if a persistent infection exists, for example, in tuberculosis, schistosomiasis, and with *Treponema pallidum*, then chronic inflammation may take over [137]. Indeed, chronic inflammation may occur from the outset. The so-called foreign body reaction and chronic inflammation are synonymous [138, 187]. Chronic inflammation involves monocytes, macrophages, lymphocytes, plasma cells and fibroblasts and is also mediated by a range of substances including the complement system, cytokines, growth factors, and reactive oxygen species. The end point is tissue destruction and fibrosis, and the condition is a disease in its own right. Granuloma formation, the aggregation of macrophages accompanied by lymphocytes, may also occur. Chronic inflammation is associated with cancer. Indeed, over recent years, that association has grown stronger and a range of tumours has now become linked with chronic inflammation in humans including carcinoma of the prostate, lung, pancreas, stomach and bowel, and other organs [188–204]. 

Chronic inflammatory bowel diseases including Crohn's disease and ulcerative colitis, as well as conditions that result in chronic inflammation such as oesophageal reflux and laryngeal inflammation, may predispose to cancer [205–209]. Sarcomas have been shown to develop at the site of chronic tropical ulcers [210]. In chickens infected with Rous sarcoma virus, a known oncogenic virus, inflammation appears to be responsible for sarcoma induction at the site of experimentally inflicted wounds, possibly mediated by TGF-*β* and other factors, and released by inflammatory cells at the site of injury [211–220]; TGF-*β* is a major mediator of fibrosis and may promote tumor development through immunosuppression and the initiation of angiogenesis, possibly through signalling involving the Smad pathway [221–224]; mice lacking Smad3 show accelerated wound healing and a reduced local inflammatory response [225, 226]. Indeed, wounds or other tissue damage may promote carcinogenesis through the involvement of growth factors regardless of the origins of the tumor [227–232]. This may involve the activation of toll-like receptors initiating inflammation [233, 234]. Chronic inflammation in the form of emphysema, possibly mediated by cytokines and chemokines, may lead eventually to lung cancer [235]. Whatever initially led to the underlying disease, the chronic inflammation may be seen as the forerunner, or even the cause of cancer.

As an example, schistosomiasis (bilharzia) is associated with the development of bladder cancer in humans [236–244]. Why should a parasitic infection lead to tumours of this nature (not to mention the intestine, liver, and uterus)? The parasites cause physical damage to the mucosa and urothelium leading to hyperplasia, but they also, possibly along with bacteria, result in chronic inflammation [218, 243]. In these cancers, chromosomal damage and mutations, including mutations in the p53 suppressor gene have been found, and these may be due to oxygen radicals persistently produced by the cells characteristic of chronic inflammation, notably by macrophages, neutrophils, and eosinophils [241, 243, 245–247].

A similar picture exists with gastric cancer in humans. It has been recognised in recent years that gastric cancer is linked to chronic infection with *Helicobacter pylori*, gastric ulceration and chronic inflammation [137, 238, 248–253]. Although the genesis of this tumor is multifactorial, chronic inflammation plays a major role [253, 254]. Carcinogenesis accompanying gastroesophageal reflux is also associated with chronic inflammation [255].

In this way, these infections may reflect similar effects noted in chemical carcinogenesis. For example, some nongenotoxic carcinogens including butylated hydroxyanisole (BHA), caffeic acid, and sesamol cause forestomach tumors in rats through irritant effects which over prolonged periods of administration result in chronic inflammation while others which produce urolithiasis, irritation, and chronic inflammation such as uracil, melamine, and sodium saccharin result in cancers of the bladder and urinary tract [256–265].

There are several other chemicals, generally regarded as nongenotoxic, which may exert carcinogenic effects through the medium of oxidative stress, chronic inflammation and reactive oxygen species. These include bile acids, silica, arsenic, and substances which lead to renal tumours in male rats through a hyaline droplet nephropathy involving *α*_2*μ*_-globulin [266–275]. DNA strand breaks have been noted in cells in the fibrotic capsule in rats following implantation of foreign bodies [187].

The association between chronic inflammation, oxidative stress, and reactive species and carcinogenesis, even in the absence of genotoxic effects, is very strong [109, 249, 276–292]. Indeed, DNA damage resulting from chronic inflammation and linked to reactive oxygen and nitrogen species, has been shown to contribute to carcinogenesis of the colon in mice [285]. DNA damage, including DNA methylation is also a direct consequence of many nongenotoxic carcinogens [286]. Consequently, a scenario can be envisaged where a foreign body (or other source) can induce acute inflammation through the intermediacy of eicosanoids or vasoactive amines and then with a failure in resolution, or *de novo* by way of tumor necrosis factor (TNF), growth factors, and other cytokines and chemokines [192, 253, 293–301] a self-perpetuating chronic inflammation ensues with responses by a number of cells and notably macrophages and fibroblasts [302–304]. As these cells then continue to destroy or phagocytose the foreign body, through *inter alia* the employment of lipoxygenases, cyclooxygenases, and other enzymes, free radicals are generated which can then result in genotoxic damage [277, 278, 282, 286, 305] possibly with the involvement of stem cells [305] and the development of tumors. Chronic inflammation may also be involved in the subsequent processes of invasion and metastasis, particularly through leukocyte infiltration and associated chemokines [188, 283, 305–307].

Oxidative stress and DNA damage induced by genotoxic and nongenotoxic agents (including TNF-*α*) activates the tumor suppressor protein p53 and other central regulatory genes [279, 308–312]. This protein has a complex range of functions including the arrest of cells in the cell cycle, permanent cell cycle arrest, and the initiation of apoptosis [308, 313–324], a process that, in addition to other functions, removes defective or damaged cells [325–328]. In human cancers, there is a high rate of mutations in the p53 gene and inactivation of p53 thus facilitates tumorigenesis in humans and in animals [329–340]. In human skin carcinogenesis, inactivation of the p53 gene and the onset of genomic instability are the earliest events in the process [341].

Hence, the p53 gene and its protein are not only major factors in cell cycle control, especially in carcinogenesis, the protein itself is an indicator and biomarker of DNA damage and mutagenesis and if suppressed, cell proliferation will ensue [45, 312, 342–349]. Some oncogenic viruses have evolved mechanisms to negate the effects of p53 [350]. Some genotoxic carcinogens may also disrupt apoptosis through disruption of growth control proteins such as c-*myc* or Bcl-2 [320, 351].

So, is there any evidence that vaccines or aluminium-based adjuvants can induce any of these events? A study of feline vaccine-associated sarcomas showed just over 51% to be surrounded by macrophages and lymphocytes with granulomatous reactions; similar indicators of chronic inflammation have been noted in other experimental studies [30, 31, 186, 352]. In dogs, injection-site sarcomas have inflammatory infiltrates including lymphocytes, macrophages, and plasma cells [181]. In an experimental study, cats treated with adjuvanted vaccine, including an aluminium-based adjuvant, had more inflammation than those treated with nonadjuvanted vaccines. Moreover, there was evidence of residual adjuvant material at the injection site [353]. Lipid-adjuvanted vaccines also resulted in inflammation in cats at the injection site [354]. In humans, aluminium adjuvants may function through stimulating dendritic cells, cells which play a major role in the process of inflammation [355].

Experimental studies show that foreign body reactions in dogs involve granulomatous reactions typical of chronic inflammation [356]. A cat which developed a sarcoma adjacent to the site of a microchip insertion had evidence of a cellular response typical of chronic inflammation [179]. Similar findings were made in dogs implanted with microchips [357], including in a dog that developed a fibrosarcoma near to a microchip insertion site [183]. Inflammation has been noted in B6C3F1 mice at the site of microchip-induced sarcomas [184]. Mild inflammation and granulomatous reactions were seen in mice implanted with microchips [358, 359]. Interestingly, microchips resulted in sarcomas at the site of microchip implantation in heterozygous p53^+/−^ mice; this was associated with increased oxidative and nitrative stress [360, 360].

In several studies, p53 protein has been detected in these feline sarcomas [65, 361–364]. Loss of p53 heterozygosity and loss of p53 function has been observed in *Trp53^+/−^* mice implanted with subcutaneous plastic plates. These mice developed sarcomas at the implantation site [360]. There was evidence of oxidative and nitrative stress in these animals as a result of implantation. Oxidative stress is associated with the process of carcinogenesis [365–370].

## 4. Discussion

In conclusion, some of the injection-site associated sarcomas seen in cats (and occasionally in dogs), as well as the microchip associated sarcomas, may be examples of foreign body tumours associated with foreign material, probably insoluble aluminium compounds (or microchips) which results in inflammation, failure of resolution, chronic inflammation, and subsequent DNA damage and cell proliferation, with the induction of p53 as a counter measure to suppress the process of carcinogenesis through disruption of the cell cycle and initiation of apoptosis. The process is similar to the sarcomagenesis seen in experimental studies with a variety of agents including food colorants, plastics, and metals. Mutations of p53 have been found in some feline and canine soft-tissue sarcomas [371–373]. Nanosized titanium dioxide has been shown to be cytotoxic to PC12 cells (derived from rat adrenal medulla pheochromocytoma) and results in oxidative stress, probably through the accumulation of reactive oxygen species, and apoptosis [374], a situation which may mimic the effects of vaccine adjuvants *in vivo*. The finding of metalloproteinase expression in feline injection-associated sarcomas supports the involvement of an inflammatory mechanism [375] a process shown to promote tumour growth in mice [376].

The situation may also be similar to that seen with asbestos-related mesothelioma in humans. Among the mechanisms envisaged for asbestos-related carcinogenicity, with ample data to support it, is chronic inflammation of the mesothelium with cytokine release, infiltration of inflammatory cells, reactive species and DNA damage and induction of p53 [377–381].

However, not all vaccines contain insoluble adjuvants and injection site sarcomas have been noted in animals after injection of pharmaceutical products. Hence, the role of insoluble material may not be critical. This raises the possibility that wounding itself, the process of injection, may be involved and, as discussed earlier, wounding and trauma, with an accompanying inflammatory process, may be involved in sarcomagenesis. None of this explains why the cat is so susceptible to the induction of these tumors or indeed, why they frequently appear in animals at a very young age. Although there is some evidence for the occurrence of these tumors in dogs, the incidence is extremely low compared with that in cats. Humans are given a variety of vaccines and injectable drugs over the course of a life-time but, apart from a few reports, there is no firm evidence to indicate that these treatments lead to tumor induction at the site of administration. All of this may suggest that the cat has a genetic predisposition to injection-site-related sarcomas, or at least to the damage related to injection which ultimately predisposes to sarcomagenesis. This may help to explain the cat's susceptibility to ocular sarcomas, some of which occur at the site of previous trauma [382–388].

Vaccines are important prophylactic tools that protect against a range of debilitating and frequently fatal diseases in animals and in humans while pharmaceutical products are vital in the treatment of disease. The low levels of risk from injection-associated side-effects must be weighed against the undoubted benefits of these products. Through the processes built into modern pharmacovigilance procedures it is important to constantly monitor these effects, including injection-site sarcomas in companion animals, and notably in cats. As has been noted recently for human vaccines, the risks and benefits must be continually evaluated [389] and this is allowed in modern pharmacovigilance practices.
